# Exosomes in head and neck cancer. Updating and revisiting

**DOI:** 10.1080/14756366.2019.1662000

**Published:** 2019-09-08

**Authors:** Samuel Rodríguez Zorrilla, Abel García García, Andrés Blanco Carrión, Pilar Gándara Vila, Manuel Somoza Martín, Mercedes Gallas Torreira, Mario Pérez Sayans

**Affiliations:** a Oral Surgery and Implantology Unit, School of Medicine and Dentistry, University of Santiago de Compostela, Santiago de Compostela, Spain;; b Oral Surgery and Implantology Unit, School of Medicine and Dentistry, Instituto de Investigación Sanitaria de Santiago (IDIS), Santiago de Compostela, Spain

**Keywords:** Exosomes, extracellular vesicles, oral squamous cell carcinoma, tumour microenvironment, head and neck cancer

## Abstract

Exosomes have gone from being considered simple containers of intracellular waste substances to be considered important carriers of cellular signals. Its broad capacity to promote tumour growth, both *in situ* and metastatic, has greatly intensified scientific research on them. In the same way and depending on its content, its tumour suppressive properties have opened a window of light and hope in the fight against cancer. In the present review we try to gather in a simple and understandable way the most relevant knowledge to date on the role of exosomes in oral squamous cell carcinoma, helping to understand their process of formation, release and activity on the tumour microenvironment.

## Introduction

The most frequent type of head and neck cancer is squamous cell carcinoma (SCC), which accounts for more than 90% of cancers of the head and neck^1^. One of the primary locations where this disease manifests is in the oral cavity; however, it occurs frequently in the pharynx and larynx as well^2^. Oral cancer is the sixth most commonly diagnosed cancer in the world and was responsible for 145, 000 deaths in 2012, 77% of which happened in regions with a poor economic development^3^. Oral squamous cell carcinoma represents 90% of all oral cancer varieties with a very common lymph node involvement^4^. The five-year survival rate for patients with localised oral squamous cell carcinoma is higher than 80%, however this rate drops dramatically to 40% when the lymph nodes are involved, and to 20% for patients with distant metastasis^5^.

Recently, numerous studies have implicated the HPV infection as a risk factor for developing oral squamous cell carcinoma, nonetheless the best described risk factors continue to be tobacco and alcohol^6^. As we have stated, cancer metastasis is related to bad prognosis and it is very frequent in these kinds of tumours, nearly 50% of these patients suffer from metastasis^7^.

In general, the extracellular vesicle population is comprised of different types of small vesicles which include microvesicles (MVs) and exosomes. Both exosomes and MVs are membrane-bound vesicles, distinguished by their biogenesis and biophysical properties, such as size and surface protein markers, and by their biological roles, including cell-cell communication, the maintenance of normal physiological processes and disease pathology^8^. The MISEV2018 position paper from the International Society for Extracellular Vesicles provided clarity on this, as well as serving as a practical guide when carrying out exosome studies^9^.

**Figure 1.**
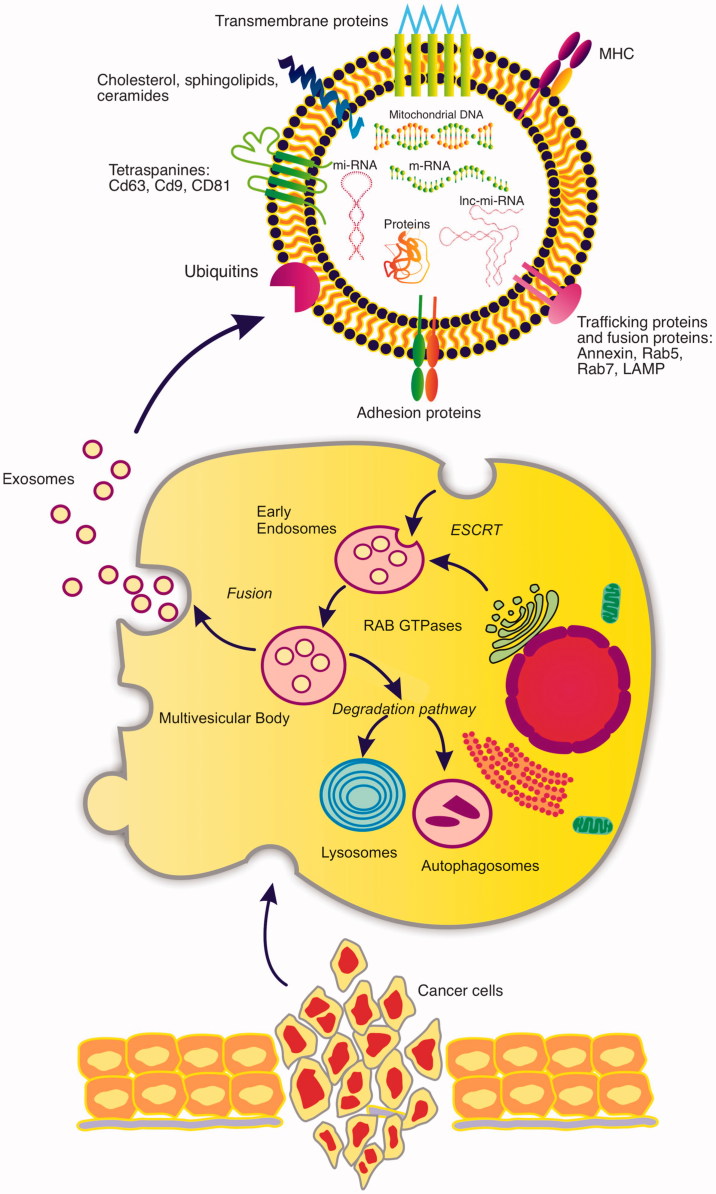
The diagram reveals the different steps of the formation of exosomes and their release from the cells of an oral squamous cell carcinoma. The intracellular traffic and its release to the extracellular medium is appreciated. In the upper image, the main components of an exosome derived from an OSCC are reflected both at the level of its lipid membrane and inside it.

Exosomes are small nanovesicles from 50–150 nm in diameter which are released into the extracellular environment, Figure 1. These are even released in biological fluids such as saliva, urine and blood^10^. Exosomes do not derive from the shedding of plasma membrane fragments, but are secreted by peculiar structures referred to as multivesicular bodies (MVBs). MVBs are the result of a cascade of multifusion phenomena among internal vesicles, including early and late endosomes, lysosomes, and other structures that vary depending on the cellular source^11^.

Exosomes were initially considered as products of a pathway which was used to release undesired material from cells^12^ and recent data support the role of exosomes, both in maintaining normal body homeostasis and in the pathophysiology of many diseases. Exosomes perform an important range of extracellular functions which include interactions with the cellular microenvironment through immunologic mediation, morphogen signalling, cell recruitment, and the horizontal transfer of genetic material^13^.

Many cell types such as dendritic cells, B cells, T cells, mast cells, epithelial cells, and tumour cells can produce exosomes^14^. Tumour cells secrete large amounts of exosomes that promote tumour progression through communication between the tumour and the surrounding stromal tissue, the activation of the proliferative and angiogenic pathways, and the initiation of the pre-metastatic site^15–19^. For these reasons, exosomes form an important component of the tumour microenvironment and are considered to be one of the main contributors to tumour progression and metastasis^13^. Exosomes’ external part is mainly comprised of lipids, while the molecules found inside, whose biological activity has been studied recently, include different proteins and RNA fragments.

As we have already stated, the main components of exosomes are lipids. Exosomes are enriched in cholesterol, diglycerides, glycerophospholipids, phospholipids, and sphingolipids or glycosylceramides^20^. Besides these lipids, bioactive lipids, such as prostaglandins and leukotrienes, and enzymes activated in lipid metabolism, including phospholipase C, are also found in exosomes^21^. Exosomes that contain high levels of prostaglandin PGE2 are involved in tumour immune evasion and in the promotion of tumour growth^22^.

Unlike MVs, exosomes do not initially carry nuclear DNA but they may contain mitochondrial DNA^23^. Valadi et al. was the first author to demonstrate the presence of mRNA and microRNA in exosome preparations^24^. MicroRNAs are small, ranging from 22 to 25 nucleotides in length. They are non-coding RNAs with the ability to interfere with RNA post-transcriptionally by binding to the 3′ untranslated region of their target mRNAs to degrade it, or suppress or stimulate translation. Their most common functions are stem cell differentiation, haematopoiesis, differentiation and organogenesis, exocytosis, and tumorigenesis^25^.

More than 3400 mRNA molecules shuttled by exosomes and 2800 microRNA are reported in ExoCarta entries, however, this is only a small fraction of the large amount of RNA contained in the exosomes.

The tumour suppressor miRNAs (TS-mi RNAs) promote the expression of tumour suppressor genes avoiding the expression of oncogenes^26^.

Exosome protein composition includes both an ubiquitary and a cell type-specific protein, and covers numerous biological functions. Most of the exosomal proteins which have been identified derive from the endocytic compartment or the plasma membrane of cellular source and only seldomly from other internal compartments such as Golgi, nucleus, endoplasmic reticulum, and mitochondria.

The most relevant protein functions carried out by exosomes in relation with the major biological function are: adhesion molecules, antigen presentation cytoskeletal/structural proteins, enzymes, heat shock proteins/chaperones, lipid raft proteins, membrane trafficking, transport and fusion, MVB formation, signal transduction, transcription/protein synthesis and transmembrane molecules^27^.

Exosomes constitutively express tetraspanins (CD63, CD9, CD81), endosomal and lysosomal markers (Rab5, LAMP), and heat shock proteins (HSP 70)^28^. These nanovesicles are completely different to apoptotic microparticles which bear markers like CD31 or annexin V^29^.

The tumour milieu is characteristically acidic as a consequence of the fermentative metabolism of glucose that results in massive accumulation of lactic acid within the cytoplasm. Tumour cells get rid of excessive protons through exchangers that are responsible for the extracellular acidification that selects cellular clones that are more apt at surviving in this challenging and culling environment. *In vitro* studies have recently pointed out that cancer acidity is a major determinant in inducing increased exosome release by human cancer cells, by showing that exosomal release was increased as the pH moved from 7.4 pH to the typical pH of cancer that is 6.5^30–32^.

A very recent study by Patton et al. demonstrated that small EV and primarly exosomes were the most bioactive in promoting the survival of hypoxic pancreatic cancer cells and hypoxia‐inducible factor‐1α stabilisation was involved in heightened EV release under hypoxia and for their potency to promote hypoxic cell survival^33^.

Through an adapted ELISA test, which allows for the detection, characterisation and quantification of exosomes, it has been demonstrated that tumour patients have significantly increased plasmatic levels of exosomes expressing CAV1 compared with the plasma of healthy donors^34^ and even CD63^35^.

A recent study has demonstrated that surgical treatment induced a dramatic reduction of the plasmatic levels of exosomes expressing CD63 as early as 1 week after resection. This first result appears to suggest that the tumour mass is responsible for the high levels of circulating exosomes detected in cancer patients^36^.

The discovery around 10 years ago that exosome contents can be transferred to another cell via fusion to create phenotypic alterations supports intensive research in this field^24^.

## Exosomes in the cancer process

Recent articles have shown that exosomes are present and involved in numerous phases of the cancer process.

It is possible to divide the aforementioned phases in a generic manner^37^: tumourigenesis, growth and development, creation of new blood vessels that feed the tumour, evasion of the immune response, development of resistance to chemotherapeutic agents and, finally, metastasis.

### *In situ* tumourigenesis

Exosomes have been defined as promoters of tumour progression^38^. Despite the fact that there is abundant *in vitro* evidence demonstrating the exchange of information between tumour cells by exosomes, in 2015 it was demonstrated, by *in vivo* techniques using a high resolution image and the Cre-LoxP system, that the exosomes released by malignant tumour cells are taken up by less malignant tumour cells which are located within the same and within distant tumours and that these EVs carry mRNAs involved in migration and metastasis^39^. Melo et al. have demonstrated how exosomes released by mammary tumour cells can cause cells from adjacent epithelial tissues to transform into tumour cells^40^.

The cancer-associated fibroblasts (CAFs) are the most abundant cells in the tumour’s immediate microenvironment. These are capable of releasing exosomes that transfer miRNAs and various proteins which accelerate the growth of these tumours^41^. It has also been shown that the tGF-B1 transported by the exosomes is capable of producing a powerful activation of the myofibroblasts, a limiting step in tumour growth and invasion^42^.

### Tumour growth

It has been understood for some time that glioblastomas release exosomes. These vesicles are rich in mRNA, miRNA and angiogenic proteins. They are taken up by normal host cells, such as brain microvascular endothelial cells and glioma cell lines stimulating aggressiveness and tumour growth^43^.

Osti et al. demonstrated the role of plasma extracellular vesicle concentration levels in glioblastoma clinical diagnosis, and in providing indications about tumour and therapy response^44^.

MET oncoproteins which are contained in exosomes can support tumour growth in hepatic carcinoma^45^. Another study referring to the same type of carcinoma, demonstrated that that the miRNA liberated in exosomes by HCC is an important mechanism for intercellular communication that can modulate TAK1 expression with the subsequent tumour growth^46^.

Li et al. demonstrated that exosomes carrying miR-1246 can be transferred among different cell lines through direct uptake and can suppress the expression level of its target gene, Cyclin-G2 (CCNG2). By this pathway the tumour volume, migration and chemotherapy resistance of these cells are increased^47^.

MiR21 is transferred from cancer-associated adipocytes (CAAs) or fibroblasts (CAFs) to the cancer cells where it suppresses ovarian cancer apoptosis and confers chemoresistance by binding to its direct novel target, APAF1^48^. In the same way, there are also exosomes with antitumour effect that compete biologically with the pro-tumoural exosomes and which can modulate the tumour growth^49^.

### Angiogenesis

The process of pathological angiogenesis is closely related to the tumour development, providing it with vessels to nourish it and giving the tumour the ability to spread to other tissues^50^. Exosome production is enhanced by intratumoural hypoxia, and endothelial cells uptake these cancer cells derived exosomes in order to stimulate the pathological angiogenesis^43^^,^^51^^,^^52^.

The aforementioned exosomes not only influence vascular growth, but can also influence their metastatic capacity. These exosomes have the ability to modify vascular fragility, making it easier to penetrate tumour cells^19^.

Endothelial cells uptake cancer-secreted miR-105 from breast cancer cells targeting the tight junction protein ZO-1, destroying tight junctions and the integrity of these natural barriers against metastasis^53^.

On the other hand, it was discovered that mesenchymal stem cell derived exosomes induce a significant and dose-dependent decrease in the expression and secretion of vascular endothelial growth factor (VEGF) through modulating the mTOR/HIF-1α signalling axis in breast cancer-derived cells^54^.

### Evasion of immune response

Recently, Razzo et al. demonstrated how a single IV injection of tumour-derived exosomes was sufficient to condition mice harbouring premalignant OSCC lesions for accelerated tumour progression in concert with reduced immune cell migration to the tumour^55^.

Currently the available evidence indicates that there is a dual role in cross communication between tumour cells and the cells of the immune system. On the one hand, it has been noted that there are several genes which stimulate the immune system, these include mesotheline and carcinoembryogenic antigen (CEA) which are released by tumour cells through exosomes^56^.

On the other hand, many studies have noted that some tumour cells release exosomes which contain proteins and nucleic acids that produce a negative regulation of the immune response^57^.

To escape destruction by the immune response, tumours avoid being recognised by cytotoxic cells, directly impair the functioning of APCs (antigen-presenting cells) or cytotoxic cells, or induce suppressor cells which consequently shut down immune reactions. Immune cells are even converted into supporters of tumour growth and survival. Exosomes participate in all these strategies through proteins exposed at their surface, and intra-vesicular cytokines and nucleic acids^58^. The most common effects of cancer-derived exosomes activity against the immune system are:

- Defective Antigen Presentation: NKG2D ligand-carrying exosomes impair NKG2D-mediated NK-cell cytotoxicity by acting as a decoy, thus contributing to immune evasion^59–61^.
- Suppression of APCs and Cytotoxic T Cells: Suppression of T cell proliferation through the expression of TGF-beta on the surface of exosomes^42^.
- Induction of the apoptosis process in T cells through the receptor-mediated pathway^62–64^.
- Regulation of T cell mRNA transcription which regulates the expression of key immune function-related genes through miRNAs carried by cancer cell exosomes^65^. Li et al. recently demonstrated that oxygen pressure in the tumour microenvironment orchestrates an anti- and protumoural γδ T-cell equilibrium by altering TEX content, which subsequently regulates MDSC (myeloid derived suppressor cells) function in a miR-21/PTEN/PD-L1-axis-dependent manner^66^.
- The extracellular adenosine release by induction of exosomes with CD39 and CD73 on their surface is a potent immune regulatory factor that protects cells and tissues from immune-mediated damage and negatively regulates local immune responses^67^. Exosomes induce pro-inflammatory activity of MSCs which in turn get tumour supportive characteristics^68^.

### Resistance to chemotherapeutic agents

The resistance of tumour cells to chemotherapeutic agents is one of the greatest challenges for its pharmacological treatment. In a heterogeneous tumour environment the way in which less aggressive cells are able to change their phenotype towards greater aggressiveness and resistance has been observed. This is due to the transfer of miRNA, mRNA and proteins released by the exosomes from one cell to another cell^69^.

Exosomes mediate a horizontal transfer of drug-resistant trait in chronic myeloid leukaemia cell by delivering miR-365^70^. In this way, it has been demonstrated that exosomes secreted by acute myeloid leukaemia (AML) cells are an essential communicator for the interaction of bone marrow stromal cells and AML which can protect AML cells from chemotherapy drug-induced apoptosis^71^.

A recent study by Qin et al. found that CAF-derived exosomal miR-196a confers cisplatin resistance in HNC by targeting CDKN1B and ING5. This finding indicates that miR-196a may serve as a predictor of, and potential therapeutic target for cisplatin resistance in HNC^72^. CAFs exposed to gemcitabine in pancreatic cancer significantly increase the release of exosomes that increase chemoresistance inducing factor, Snail, in recipient epithelial cells and promote proliferation and drug resistance^73^. Exosomes can mediate taxol resistance by upregulating Septin-9 in hepatic cell carcinoma^74^, sunitinib resistance in renal cancer^69^, and cisplatin resistance in lung cancer^75^.

Exosomes can also play an important role in drug effluxion by encapsulating and exporting the drugs from inside to outside, avoiding the therapeutic effect of those^76^. Moreover, exosomes regulate the binding of antibodies to cancer cells, therefore playing an important role in downregulating their therapeutic effect.^37^.

### Metastasis

Metastasis is the first cause of death for a large majority of cancer patients despite the advanced techniques in radiotherapy, chemotherapy, immunotherapy and advanced surgical techniques^77^.

Recent data have been shown that EVs and specially exosomes may transform the microenvironment of primary tumours thus favouring the selection of cancer cells with a metastatic behaviour^78^. The release of exosomes and other EVs from resident non-malignant cells may contribute to the metastatic processes as well. However, cancer EVs may induce malignant transformation in resident mesenchymal stem cells, suggesting that the metastatic process is not exclusively due to circulating tumour cells^79^.

Metastatic niche refers to the creation of a microenvironment in different organs and tissues that allows for the nesting and development of DTCs (disseminated tumour cells^80–83^.

The available scientific evidence indicates that metastatic niches are initiated previously (pre-metastatic niches) through the interaction of factors secreted by tumour cells, the recruitment of tumour progenitor cells, the recruitment of haematopoietic progenitor cells (HPC), myeloid cells, and mesenchymal stem cells (MSC) of the bone marrow, which allows for DTC nesting and subsequent growth, which depends on endothelial precursor cells (EPC) and finally on angiogenic factors^84^.

Modulation of vascular permeability and the stimulation of neo-angiogenesis are key steps during pre-metastatic niche formation which favours the initial extravasation and subsequent metastatic growth of tumour cells in secondary organs^81^.

Oral squamous cell carcinoma hypoxic microenvironment may stimulate tumour cells to release miR-21–rich exosomes that are delivered to normoxic cells to enhance prometastatic behaviour^85^.

Silencing of the miR-200c-3p targets, CHD9 and WRN, significantly accelerated the invasive potential of SQUU-A cells in squamous tongue carcinoma. miR-200c-3p in exosomes derived from a highly invasive OSCC line can induce a similar phenotype in non-invasive counterparts^86^.

A recent paper by Zhou et al. demonstrates that proteomic exosomes cargo PF4V1, CXCL7, F13A1 and ApoA1 from serum is related to the metastasis of OSCC^87^.

Despite last years and recent discoveries, there still lies a gap in our knowledge of the dynamics of exosomes mediated metastatic processes: from the formation of the pre-metastatic niches, to the metastatic niches to the actual formation of the metastatic lesion.

What we clearly observe is that they play a key role in the preparation of the metastatic niche long before the arrival of any cancer cell in the area, even showing certain exosomes affinity for certain organs for the preparation of the metastatic niche.

## The role of exosome in oral squamous cell carcinoma

The effect of exosomes released from oral squamous cell carcinoma (OSCC) into the tumour microenvironment and distant metastasis process is still not completely clear. It has been demonstrated that OSCC cell-derived exosomes are taken up by OSCC cells themselves and significantly promote proliferation, migration, and invasion through, among others, the activation of the PI3K/Akt, MAPK/ERK, and JNK-1/2 pathways *in vitro*^88^.

Recent studies have shown an aberrantly expressed pattern of miRNA identified in both tumour and plasma of patients with OSCC, suggesting that this may be a biomarker for OSCC. It has become apparent that aberrations within the noncoding genome drive fundamental cancer phenotypes in addition to the best-known protein coding mutations^89^. Circulating exosomes appear to be a reliable method for evaluating circulating tumour-miRNA expression^90^. Multiple OSCC cell types exist in a single tumour mass and these secrete exosomes containing a unique set of miRNAs. Through the use of two unique malignant cell clones and by analysing the exosome-derived miRNAs, it was demonstrated that miR-200c-3p is an oncogenic miRNA which is capable of inducing invasive potential in non-invasive cells within an OSCC tumour mass^86^. On the other hand, exosomes derived from mesenchymal stem cells of human bone marrow that overexpress mi-RNA-101-3p suppress the proliferation, invasion and migration of oral cancer cells. Said mi-RNA is under-expressed in the cells of oral squamous cell carcinomas^91^.

PCR based array methods identified the role of miRNA-26a and miRNA-26b in OSCC that enhances cancer cell migration and invasion through regulation of TMEM184B^92^. As we stated, miRNAs are detected in the extracellular vesicles in OSCC; for example miRNA-21 was detected in exosomes derived from OSCC under hypoxic conditions. Snail and vimentin expression was significantly enhanced while the E-cadherin levels were decreased both *in vitro* and in vivo studies. Moreover, circulating exosomal miRNA-21 levels were associated with HIF-1α/HIF-2α expression, T stage, and lymph node metastasis in patients with OSCC. These findings suggest that the hypoxic microenvironment may promote prometastatic behaviours, stimulating tumour cells to generate miRNA-21-rich exosomes that are delivered to normoxic cells^85^.

OSCC exosomes are important intercellular communicators, delivering proteins, mRNA and miRNA to selected targets during premetastatic niche. The enrichment of tetraspanins within secreted exosomes hints the role of tetraspanin in the regulation of exosome uptake. Profiling of exosomal CD151 web proteins in the unexplored process of selection and uptake may lead to the early detection of cancer. Exosomal biomarker-based diagnosis is also useful both in laboratory research and clinical medicine, suggesting exciting new directions for future research. Tetraspanins are potential targets for drug development, not only in the area of neoplasias, but also in infectious diseases, given that many tetraspanins are known to facilitate infection processes of various pathogens, for example viral, bacterial and protozoan infections^93^.

Overexpression and increased signalling from the epidermal growth factor receptor (EGFR) often change oral squamous cell carcinoma (OSCC) and thus EGFR is frequently targeted molecularly by the therapeutic antibody cetuximab. Fujiwara et al. assessed the roles of OSCC-derived extracellular vesicles (EVs) including exosomes in the trafficking of cetuximab and in epithelial-mesenchymal transition (EMT) of epithelial cells. OSCC cells abundantly expressed EGFR, which was secreted from cells with OSCC-EVs upon EGF stimulations. The OSCC-EGFR-EVs were then able to enter into and transform epithelial cells leading to increased mesenchymal traits with increased vimentin and spindle-like shapes. EGF priming of OSCC cells further increased this EMT-initiating effect of the OSCC-EVs. The internalisation and pro-EMT effects of the OSCC-EVs were largely blocked by cetuximab. Thus OSCC-derived EVs transform normal epithelial cells into a mesenchymal phenotype and anti-EGFR therapeutic antibody cetuximab inhibits such a carcinogenic effect of the OSCC-EVs^94^.

Data indicate that miR- 200c-3p in exosomes derived from a highly invasive OSCC line can induce a similar phenotype in non-invasive counterparts^86^. On the other hand exosomes derived from cisplatin-resistant OSCC cells were found to enhance the chemoresistance of OSCC cell and reduce the DNA damage signalling in response to cisplatin^95^.

Chen et al. demonstrated that exosomes released from HIV-infected T cells and those purified from blood of HIV-positive patients stimulate proliferation, migration and invasion of oral/oropharyngeal and lung cancer cells. The HIV transactivation response (TAR) element RNA in HIV-infected T-cell exosomes is responsible for promoting cancer cell proliferation and inducing expression of proto-oncogenes and Toll-like receptor 3 (TLR3)-inducible genes^96^.

As we stated previously, EVs are detectable in significantly higher quantities in the plasma of patients with OSCC. Oncogenic miRNAs (such as miR-21, miR-27) were detectable in high quantity in the circulating EVs and plasma of patients with OSCC. EVs were taken up by monocytes after co-culture. Mechanistically, uptake of EVs derived from oral cancer cells by monocytes caused activation of the inflammatory pathway, NF-κB activation, and establishment of a pro-inflammatory and protumourigenic microenvironment^89^.

Exosomes derived from cancer cells may express surrogate oncogenic markers such as CEP55 membrane protein and carry FOXM1 mRNA cargos. CEP55 protein in saliva or blood could be exploited as a cancer biomarker for non-invasive mode of diagnosis and prognosis of HNSCC^97^.

Tumour-derived exosomes (TEX) accumulate in the tumour microenvironment (TME) and serve as a communication system between tumour and normal stromal cells. TEX promote angiogenesis and drive HNSCC progression^98^.

Mesenchymal stem cells (MSCs) are a major component of the tumour microenvironment (TME) and play a key role in promoting tumour progression. The tumour uses exosomes to co-opt MSCs and re-program their functions. The MSCs re-programmed by TEX become avid producers of their own exosomes that carry and deliver mRNA and miRNA species as well as molecular signals not only back to tumour cells, directly enhancing their growth, but also horizontally to fibroblasts, endothelial cells and immune cells by enhancing their pro-tumour functions. TEX driven cross-talk of MSCs with immune cells blocks their anti-tumour activity and/or converts them into suppressor cells. MSCs re-programmed by TEX mediate pro-angiogenic activity and convert stromal cells into cancer-associated fibroblasts (CAFs)^99^. In this way Wang et al. recently revealed that CAF-derived exosomes contain lower miR-3188 levels than normal fibroblasts, and the loss of miR-3188 in exosomes contributes to the malignant phenotypes of HNC cells through the derepression of BCL2. Furthermore, these data suggest the potential therapeutic value of exosomal miR-3188 for inhibiting HNC growth^100^.

It is important to mention long non-coding RNAs that are functionally defined as transcripts bigger than 200 nt in length with no protein-coding potential,^101^^,^^102^, these exert their functions by affecting chromatin remodelling, transcriptional activation or suppression, miRNA sponge and miRNA splicing regulation^103^. The nc-RNAs below are among those collected by some authors that are up and down-regulated in oral squamous cell carcinoma and his proposed function in literature (Table 1).

**Table 1.** The table is divided into two sections. In the upper section, some of the most important lnc-RNAs collected in the literature appear up-regulated in oral squamous cell carcinomas as well as their main biological effects. In the lower section are those that are down-regulated in the tumours mentioned above.

Up-regulated NC-RNAs	Function
Up-regulated NC-RNAs	
MALAT1	EMT-mediated cell migration and invasion via regulating N-cadherin, Vimentin and E-cadherin^104^^,^^105^.
PANDAR	Promoter of CDKN1A antisense DNA damage activated RNA^106^.
TUC338	Enhances proliferation and reduced apoptosis^107^.
lincRNA-ROR	Acts as a sponge for miRNA-145-5p to modulate c-Myc, Kl, Sox2, and Oct4 genes^106^.
POU3F3	Regulates cell proliferation, and apoptosis^106^.
FTH1P3	Acts as a molecular sponge for miRNA-224 to modulate frizzled 5 Expression^108^.
UCA1	Promotes tumour invasion and metastasis possibly through WNT/β-catenin pathway^109^.
CCAT1	Acts as a sponge for miRNA-155-5p and let7b-5p. May be a predictor for poor treatment response^106^.
LINC00152	Correlated with cancer progression, advanced stage, cancer relapse, and invasion^110^.
AC132217.4	Promotes cell migration and EMT via IGF2 levels^111^.
MIR31HG	HIF-1α co-activator^112^.
LINC00668	Acts as CeRNA for miRNA-297 to regulate VEGFA regulation^113^.
Inc-sox5	Regulates apoptosis and cell cycle^114^.
LNC00673	Promotes tumour invasion and metastasis^115^.
miR-8485	Promotes the proliferation, migration and invasion of tumour cells^116^.
miR‑382‑5p	Induce cell migration and invasion. CAF‑OSCC communication vehicle^117^.
Down-regulated NC-RNAs	
miR-145-5p	Pro-apoptosis related miRNA^118^.
NKILA	Inhibits the phosphorylation of IKβα NF-kβ, and inhibits EMT^119^.
MEG3	Regulates cell proliferation, cell cycle and apoptosis. Therapeutic target for OSCC^120–122^.
miR-101-3p	Regulates cell proliferation by COL10A1 gene^91^.
miR-3188	Contributes to the malignant phenotypes of HNC cells through the derepression of BCL2^100^.

Recent research has made significant progress, overcoming major barriers for using exosomes as a delivery system. Exosomes are ideal systems for delivering cancer therapeutics, due to their size, surface expression profiles, low immunogenicity, low cytotoxicity, and long-term safety. Their use has opened a new promising avenue for cancer treatment^123^. A new study by Rosenberger et al. demonstrated that exosomes secreted by menstrual mesenchymal stem cells have a significant antitumour effect in the intra-tumoural injection of exosomes with a loss of tumour vasculature. The authors of this study claim that menstrual stem cell exosomes are potential anti-angiogenic agents for the treatment of neoplastic conditions^124^.

The exosomes whose content is Cav1, CD63, Rab5B and Annexin II are the most commonly described in oral cancer research^125^. The caveolin-1 gene is located at D7S522 locus of human chromosome 7q31.1. CAV1 is expressed in most cell types^126^ and is present in a variety of cellular and extracellular compartments. CAV1 has a role in both normal and pathological tissue, where it has been shown to be upregulated by the hypoxia-inducible factor (HIF)-α^127^ that enhances the oncogenic potential of tumour cells by increasing the cell’s proliferative, migratory, and invasive capacities^128^ and even chemotherapy and radiotherapy resistance^129^. Some studies on CAV1 in oral squamous cell carcinoma (OSCC) showed an increased immunoexpression of CAV1 in SCC tissue when compared to normal mucosa and precancerous (dysplastic) lesions^130^), and it was even found to be significantly over-expressed in OSCC compared to normal oral mucosa (*p* = 0.002 and *p* = 0.033, respectively) when using immunohistochemistry to demonstrate that patients with over-expression of Cav-1 protein were associated with poor prognosis (*p* = 0.030)^131^. These studies have shown a gradual increased expression of CAV-1 in the different steps of cancerous process in oral cancer^132^. Authors suggest that accumulation of CAV1-TME in TSCC had a negative prognostic value in vivo^133^. This was demonstrated in the study by Rodríguez et al. where the negative relationship between plasmatic CAV1 exosomes and the overall survival of OSCC patients was verified^36^. Vered et al. demonstrated how the accumulation of CAV1 protein in tumour microenvironment in patients with tongue squamous cell carcinoma had a negative prognostic value and how CAV1 is involved in fibroblast undergoing trans-differentiation to CAFs^134^. It is important to note that some authors propose that CAV1 does have a role as a tumour suppressor and that there is a connection between loss of Cav-1 expression and the ability of cells to escape from anchorage growth control^135^. The literature suggests that Cav-1 is downregulated in colon cancer, breast cancer, ovarian carcinoma, and soft tissue sarcomas, while increased expression was seen in ductal adenocarcinoma of the pancreas, prostate cancer, squamous cell carcinoma, glioblastoma, non-small cell lung carcinoma (NSCLC) and renal cell carcinoma^136^.

On the other hand, Western blot analysis confirmed the presence of the known exosome marker CD63^85^ in OSCC patients. Although CD63 has been originally described as a tumour suppressor^137^ reassessing its function is worthwhile as it was identified as a receptor of tissue inhibitor of metalloproteinases-1 (TIMP-1)^138^ which may promote metastasis^139^.

It is important to note that authors such as Shinya et al. have demonstrated that the uptake of exosomes by OSCC cells and subsequent tumour progression was abrogated in the presence of heparin, and for that reason it may be useful for treatment of these patients^88^. In the aforementioned study by Rodríguez et al., a negative survival rate was observed among patients with OSCC in stage IV with plasmatic levels of CD63+ exosomes higher than the mean, when compared to the survival of those patients with levels below the mean. A significant reduction of CD63 exosome levels after tumour resection was also observed, thus proving the existence of a clinical relationship as tumour markers of important prognostic value in OSCC patients and its utility in the clinical follow-up of cancer patients^36^.

### Exosomes in saliva

Salivaomics integrate the study of saliva and its constituents, functions, and related techniques^140^. The first authors to report the presence of exosomes in saliva were Ogawa et al. in the year 2008^141^.

The collection of exosomes in saliva is simple and non-invasive. It also contains less proteins than blood and therefore its identification and quantification is simplified considerably^142^^,^^143^. It has been demonstrated that they can be stored at 4° C without the need for them to be supercooled at −80° C, making it much easier to work with them in clinical environments^144^.

In the year 2011, Ogawa et al. described two types of salivary exosomes that differ mainly in terms of their size and protein composition. Type I exosomes are larger and denser to electrons than type II exosomes; however, the latter are more similar to those present in other fluids and body media^141^. Both types of exosomes contain protein markers such as CD63, Alix, Tsg101 and Hsp70, immunoglobulin A and polymeric immunoglobulin receptors, although the specific protein composition is different. Salivary human exosomes participate in the catabolism of biopeptides and play a very important role in the local immune response of the oral cavity. It is believed that the secretion of type I or II exosomes depends, to a large extent, on the type of salivary gland that produces it^145^. The Next-Generation Sequencing Technology has recently allowed for more exhaustive investigations regarding RNAs that encode long proteins to be carried out. These RNAs form an important part of the salivary exosomes and are able to translationally control tumour protein, likewise they play an important role in cell proliferation and death as well as in the immune response^146^. In 2016, Ogawa et al. demonstrated, *in vitro*, how the content of RNAs and other pseudogenes included in the interior of the salivary exosomes can be transferred horizontally to other cells, modulating the genetic expression of the receptors^147^ and increasing their invasion and migration capacity^85^.

J. Kim et al. checked the amount and type of extracellular vesicles in plasma and saliva in melanoma mice. The authors found that only 38.22% ± 18.55% of the vesicles found in plasma appeared in saliva, unlike the cases in which the tumour pathology is in the oral cavity and the exosome load is much higher due to the direct contact between the saliva and the tumour^148^.

Several of the exosomal miRNA secreted solely by cancer cells in culture were detected at substantially elevated levels in saliva from HNSCC patients compared to saliva from healthy controls. These findings provide important insight into tumour biology and yield a promising set of candidate HNSCC biomarkers for use with non-invasive saliva samples^149^.

Zahran et al. reported a highly significant increase in salivary miRNA-21 and miRNA-184 in saliva of OSCC patients when compared to healthy and disease controls and in fact, the only microRNA they found to discriminate between OSCC and oral potentially malignant disorders was miRNA-184^150^.

Sharma et al. showed that exosome size, exosome population, and inter-exosome are increased in the saliva of patients with oral cancer. Interestingly, oral cancer exosomes exhibited significantly increased CD63 surface densities and displayed irregular morphologies^151^.

Zlotogorski-Hurvitz et al. were able to show morphological as well as molecular differences in salivary exosomes of patients with oral squamous cell carcinoma with respect to healthy patients as a screening measure in patients with a high risk of oral squamous cell carcinoma^152^.

In tumour cell hypoxic environment, as in the case of oral squamous cell carcinoma, mi-RNA is produced more intensely, this is one of the genetic materials which is most easily identifiable in exosomes and it is of vital importance due to its capacity to regulate the tumour microenvironment^153^. miR-21 is one of the most intensively produced RNA genes and it promotes metastatic cell behaviour through its interaction with (PTEN) and (PDCD4), as it transmits tumour-resistant characteristics to chemotherapeutic agents such as cisplatin^95^. Releasing miR-200c-3p facilitates tumour invasion in tumour-free areas^86^^,^^154^.

Langevin et al. used next-generation sequencing for miRNA, thus differentiating miRNA from HNSCC cells in relation to healthy epithelial cells. They observed that a large amount of mi-RNA was shared in exosomes from healthy and cancerous cells. However, significantly higher concentrations of miR-486-5p, miR-486-3p, and miR-10b-5p were detected with respect to the control group^149^.

Some studies by Gai et al. detected by charged based precipitation de presence of miR-412-3p, miR-512-3p, miR-27a-3p, miR-494-3p up-regulated in the saliva of OSCC patients and miR-302b-3p, miR-517b-3p expressed only in exosomes from the saliva OSCC patients^155^^,^^156^.

As in the blood, CD63 + exosomes were also found in saliva^151^^,^^152^ that have shown prognostic value in patients with OSCC when they are counted in serum prior to and after tumour resection surgery^36^. At the same time exosomes PPIA + were detected down-regulated as a poor prognosis factor in the saliva of OSCC patients^157^.

A growing body of saliva-exosomics study is highlighting the role of cancer-derived exosomes in saliva. The properties of cancer-derived exosomes in saliva are attracting the attention of scientists since these exosomes could be used as diagnostic biomarkers, potential surrogate markers for other physical conditions, or novel immune regulatory systems through the gastrointestinal tract. However, the utility of salivary exosomes as biomarkers of diseases and conditions requires further investigations due to the current paucity of studies in this emerging area they have a promising role in next year’s investigation and will represent an important future challenge in diagnosis, treatment and prognosis of different cancer type not only OSCC due the capacity of different body cancers exosomes to be secreted by salivary glands^158^^,^^159^.

## Conclusion

In conclusion, we are able to determine that the information available in this field is limited and very recent. Most of it focuses on the quantification and qualification of the exosomes content present in plasma or saliva. However, information about its biological role with respect to squamous cell carcinoma of the mouth is small, although it increases rapidly. As mentioned, there are studies that are able to show their direct relationship with the aggressiveness of the cancer. Also its capacity as a biomarker of the presence of the disease and life prognosis of the patients who suffer it is of great relevance in the diagnosis and treatment of patients with oral squamous cell carcinoma as well as other types of cancers. More research is required in a field that is gaining considerable ground due to its importance in the carcinogenic process.
